# Etoposide-Bevacizumab a new strategy against human melanoma cells expressing stem-like traits

**DOI:** 10.18632/oncotarget.9939

**Published:** 2016-06-09

**Authors:** Maura Calvani, Francesca Bianchini, Maria Letizia Taddei, Matteo Becatti, Elisa Giannoni, Paola Chiarugi, Lido Calorini

**Affiliations:** ^1^ Department of Experimental and Clinical Biomedical Sciences, University of Florence, 50134, Florence, Italy; ^2^ Tuscany Tumor Institute and “Center for Research, Transfer and High Education DenoTHE”, 50134, Florence, Italy

**Keywords:** human melanoma A375 and Hs294T lines, cancer stem-like trait, CD133/VEGF-R2+ melanoma cells, etoposide-bevacizumab cooperation

## Abstract

Tumors contain a sub-population of self-renewing and expanding cells known as cancer stem cells (CSCs). Putative CSCs were isolated from human melanoma cells of a different aggressiveness, Hs294T and A375 cell lines, grown under hypoxia using “sphere-forming assay”, CD133 surface expression and migration ability. We found that a cell sub-population enriched for P1 sphere-initiating ability and CD133 expression also express larger amount of VEGF-R2. Etoposide does not influence phenotype of this sub-population of melanoma cells, while a combined treatment with Etoposide and Bevacizumab significantly abolished P1 sphere-forming ability, an effect associated with apoptosis of this subset of cells. Hypoxic melanoma cells sorted for VEGF-R2/CD133 positivity also undergo apoptosis when exposed to Etoposide and Bevacizumab. When Etoposide and Bevacizumab-treated hypoxic cells were injected intravenously into immunodeficient mice revealed a reduced capacity to induce lung colonies, which also appear with a longer latency period. Hence, our study indicates that a combined exposure to Etoposide and Bevacizumab targets melanoma cells endowed with stem-like properties and might be considered a novel approach to treat cancer-initiating cells.

## INTRODUCTION

Metastatic melanoma represent one of the most aggressive skin cancer, refractory to treatment due to high resistance to chemotherapy. Indeed, a 5-year survival rate of advanced stage malignant melanoma is less than 10% and resistance to chemotherapy, most likely due to the presence of cancer stem cells (CSCs), is the major cause of treatment failure [1–3]. Several studies have demonstrated that tumors contain a small subset of cells that share many characteristics with CSCs [4]. They constitute a pool of self-renewing cells with the ability of sustaining tumor growth and promoting metastatic dissemination. The symmetric division of CSCs allows cell population expansion while maintaining their distinctive traits [5, 6]. Drug resistance is probably the most important clinical feature of CSCs, allowing them to play a major role in cancer relapse, thereby representing a promising target to counteract tumor progression and recurrence [7].

Putative CSCs are isolated using methods based on several *in vitro/in vivo* assays, such as expression of distinct surface markers or intracellular enzyme activities, “sphere-forming” ability in non-adherent culture and/or initiation of new tumor growth when xenotransplanted into immunodeficient mice [8]. Evidences support the presence of CSCs in several malignancies, including those of blood, brain, breast and, recently, melanoma [9]. Melanoma show phenotypic heterogeneity both *in vitro* and *in vivo*, suggesting an origin from a cell with multi-lineage differentiation ability [10–12]. Moreover, Quintana et al. found that transplanting one melanoma cell into NOD/SCID Il12rg−/− mice gives rise to a tumor in 27% of the cases, indicating that a large population of melanoma cells is able of repopulating a new tumor, acting as CSC [13].

Malignant melanoma is a high vascular tumor with constitutive over-expression of vascular endothelial growth factor (VEGF) and VEGF receptors (VEGFRs), both associated with poor prognosis [14–16]. Indeed, VEGF-R1/VEGF-R2 expression characterizes metastatic melanoma cells [17]. Strategies to abrogate angiogenesis by inhibiting multiple signaling pathways are attractive and Bevacizumab, a humanized monoclonal antibody that neutralizes VEGF activity [18–21], and Sorafenib, an oral tyrosine kinase inhibitor with multiple targets, including VEGFRs [22, 23], are considered very interesting agents. However, contrasting results emerge from literature, e.g. Adamcic et al. [24] reported a negative effect of Bevacizumab on malignant melanoma, whereas Curtarello et al. [25] showed a clear effect of Bevacizumab on melanoma cells, although on the poorly “aerobic glycolytic” subpopulation.

Recently, we demonstrated that hypoxia induces an autocrine aggressive loop in Hs294T metastatic melanoma cells involving VEGF and VEGF-R2, and Bevacizumab and VEGF-R2 neutralizing antibodies limited the survival of hypoxic Etoposide-resistant cells [26]. Etoposide is a well-known cytostatic drug acting through apoptotic mechanisms. Treatment with Etoposide is more frequent in lung cancer, leukemia and testicular tumors, but it has been also used in combination with cisplatin for the treatment of melanoma brain metastases [27]. However, upon Etoposide plus cisplatin exposure, melanoma cells switch to a drug-resistance phenotype, associated with an apoptotic deficiency [28].

Now, using Hs294T and A375 melanoma cell lines, respectively derived from a human metastatic and primitive lesion, we demonstrate that a concomitant exposure to Etoposide/Bevacizumab eradicates a sub-population of melanoma cells endowed with stem-like features. Our finding also highlights efficacy of Bevacizumab on melanoma cells expressing stem cell markers including a functional loop of VEGF/VEGFR2.

## RESULTS

### VEGF-R2 as a target of hypoxic melanoma stem-like cells

**Figure 1:**
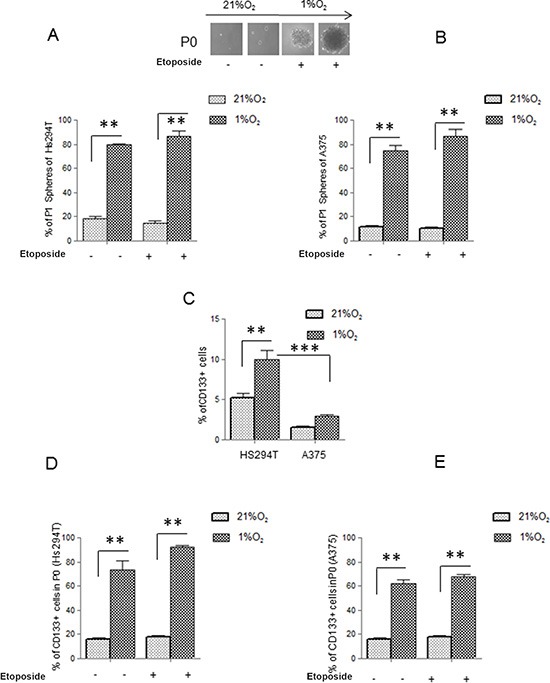
Hypoxia endows Hs294T and A375 melanoma cells with stem-like traits (**A, B**) Starved Hs294T *(left)* and A375 *(right)* cells were cultured under normoxic (21% O_2_) or hypoxic conditions (1% O_2_) for 24 h in the presence or absence of Etoposide (50 μM). Serum deprived cells in normoxic conditions (21% O_2_) were used as control. P0 (primary) and P1 (secondary) melanospheres were obtained from the above treated cells. Photographs of P0 spheres were taken and shown (above the corresponding treatment), while P1 spheres were counted and plotted. (**C**) Starved Hs294T and A375 cells cultured under normoxic or hypoxic condition for 24 hours and treated or not with Etoposide (50 μM). were analyzed for CD133 staining. (**D, E**) Cells disaggregated from P0 spheres derived from Hs294T *(left)* and A375 *(right)* cultured under normoxic or hypoxic conditions treated or not with Etoposide (50 μM). were analyzed for CD133 staining. *P* values of ≤ 0.05 were considered statistically significant *p < 0.05, **p < 0.001, ***p < 0.0001, *n* = 3.

**Figure 2:**
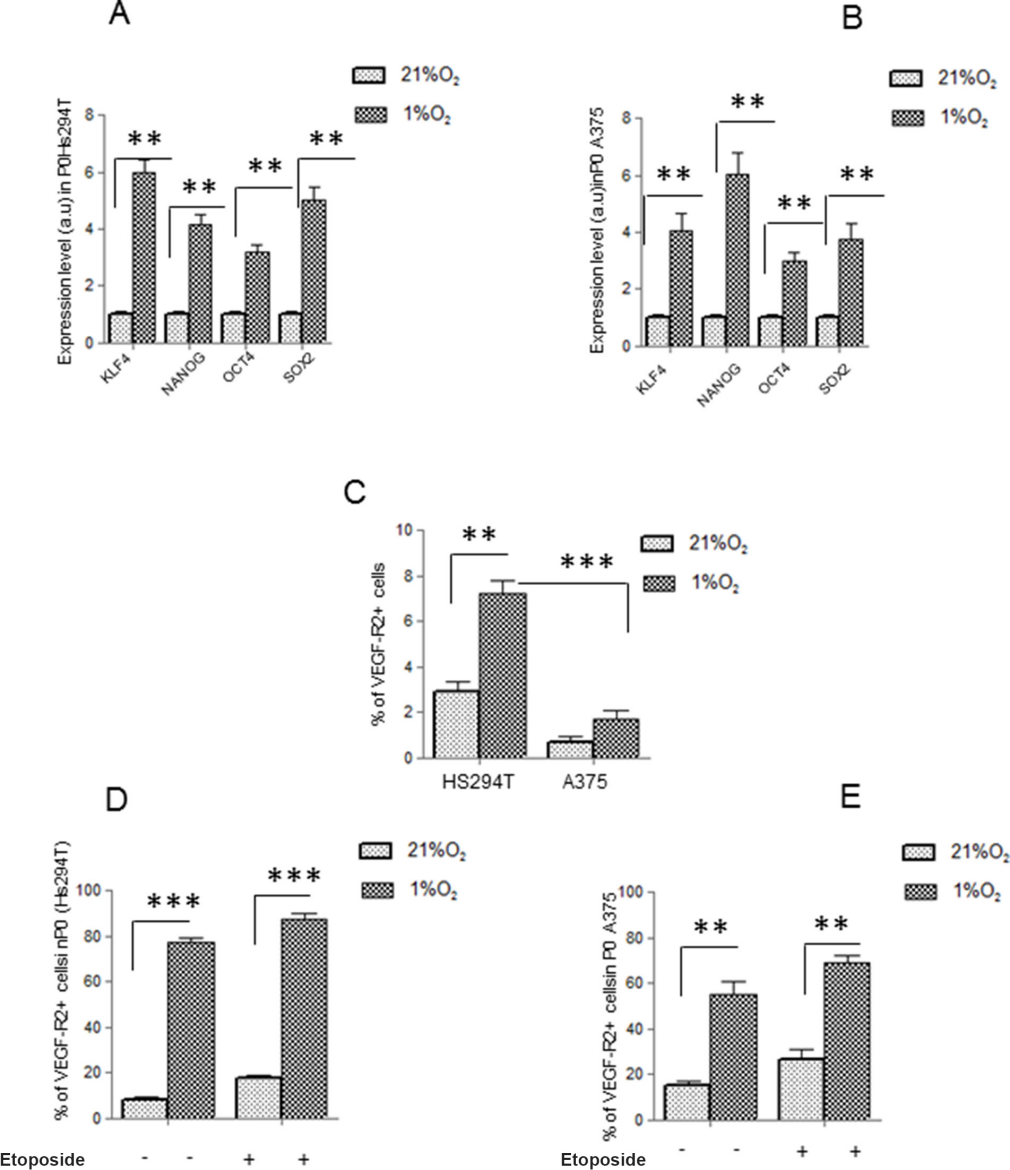
VEGF-R2 and stem cell like markers are expressed on melanoma cells (**A, B**) mRNA evaluation of KLF4, NANOG, OCT4, SOX2 was performed in P0 spheres derived from Hs294T *(left)* and A375 *(right).* Values are reported as fold change with respect to relative normoxic samples. (**C**) Starved Hs294T and A375 cells were cultured under normoxic or hypoxic condition for 24 hours and VEGF-R2 expression was assessed by flow cytometry (**D**, **E**) P0 spheres derived from normoxic and hypoxic Hs294T *(left)* and A375 *(right)* cells were disaggregated and VEGF-R2 expression was analyzed by FACS analysis. *P* values of ≤ 0.05 were considered statistically significant *p < 0.05, **p < 0.001, ***p < 0.0001, *n* = 3.

Maintenance of metastatic Hs294T and primary A375 human melanoma cells under hypoxic condition (1% O_2_) selected a subpopulation endowed with a stem-like phenotype, as indicated by their increased ability to give rise to melanospheres (primary spheres, P0) in non-adherent conditions, whereas cells grown in normoxia gave spheres in a very small amount. Furthermore, melanoma cells from P0 spheres, whether separated into single cells, are endowed with higher ability to generate secondary spheres (P1) compared to normoxic control in both type of melanoma cells (Figure 1A, 1B). Moreover data revealed that cells derived from disaggregated hypoxic P0 melanospheres were enriched in expression of CD133 (Figure 1D, 1E), a well-accepted marker of melanoma CSCs [29–34]. However, it is noteworthy to consider that hypoxic adherent Hs294T melanoma cells express higher level of CD133 compared with that of A375 cells (Figure 1C). Etoposide (LC50 20 μM) treatment did not modify, number of P1 spheres and CD133 expression in P0 melanospheres, both in metastatic and primary melanoma cells from (Figure 1A, 1B–1D, 1E). The P0 spheres derived from Hs294T cells showed a bigger dimension above those derived from A375 cells, of about 1.3 fold change. Stem cells like phenotype was confirmed by hypoxic increased mRNA expression of NANOG, KLF4, OCT4 and SOX2 in both Hs294T and A375 cells collected from P0 spheres (Figure 2A, 2B) [32, 33]. Further, hypoxic melanoma P0 Hs294T and A375 cells show an increased VEGF-R2 expression (Figure 2D, 2E), and Etoposide treatment did not modify level of VEGF-R2 in cells derived from P0 spheres of both cell line analyzed (Figure 2D–2E). As reported before for CD133 expression, also the expression level of VEGF-R2 in Hs294T is higher than that of A375 cells (Figure 2C).

**Figure 3:**
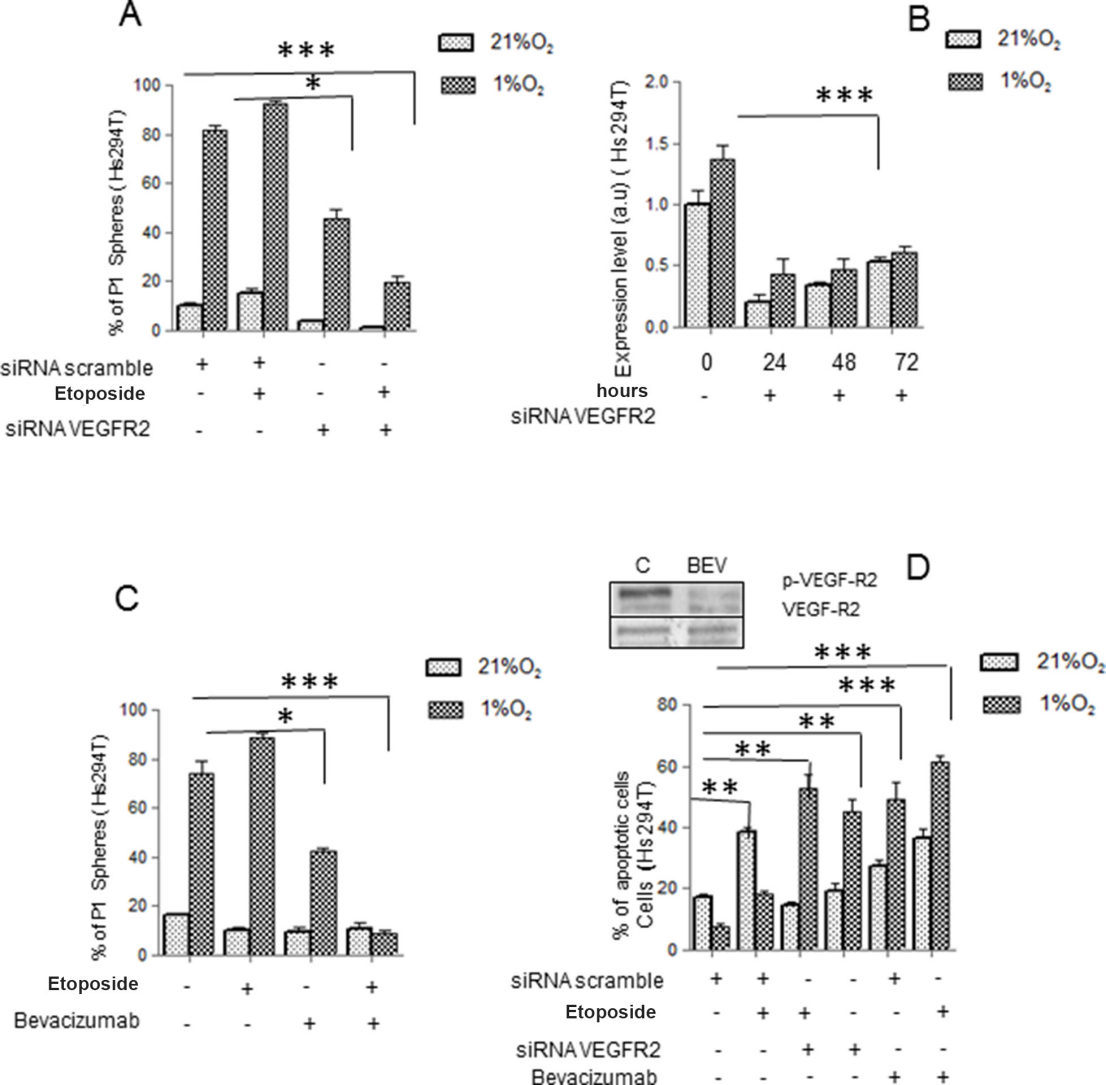
VEGF-R2 targeting abolishes hypoxic stem-like melanoma cells (**A**) Hs294T cells were silenced with siRNAs against VEGF-R2 or with scramble siRNA as control. 48 hours after transfection, cells were treated with or without Etoposide (50 μM) under normoxic or hypoxic conditions for additional 24 hours. Melanosphere formation assay was performed and P1 spheres were quantified. (**B**) VEGF-R2 silencing was confirmed by Real Time PCR under normoxic or hypoxic condition at 24, 48, and 72 hours. Serum deprived cells in normoxic conditions were used as control. (**C**) Hs294T cells were serum starved for 24 hours and then incubated in the presence or absence of Etoposide (50 μM) and/or Bevacizumab (250 ng/ml) for 24 h. Melanosphere formation assay was then performed and P1 spheres were quantified. (**D**) Apoptotic cell death of Hs294T cells treated or not with Etoposide (50 μM) and/or VEGF-R2 siRNA, and/or Bevacizumab for 24 hours, was assessed by Annexin-V/propidium iodide staining. Phosphorylation of VEGF-R2 was analyzed on Hs294T cells treated or not with Bevacizumab (250 ng/ml) under normoxic or hypoxic condition. *P* values of ≤ 0.05 were considered statistically significant *p < 0.05, **p < 0.001, ***p < 0.0001, *n* = 3.

Now, we have analyzed the role of VEGF-R2 and potential benefit of Bevacizumab use in reducing cancer stem-like cell phenotype in Hs294T cells. Indeed, Hs294T cells represent a better model of stemness compared with A375 cells. Further, as previously reported impairment of VEGF/VEGF-R2 signaling by Bevacizumab increased apoptosis rate in Hs294T by reducing reactive oxygen species (ROS) derived from NADPH oxidase [26]. In Hs294T cells, VEGF-R2-silencing promotes an impairment of P1 sphere-forming ability, an effect particularly evident when Etoposide treatment was associated (Figure 3A). We confirmed the efficacy of VEGF-R2 siRNA by Real Time PCR until 72 hours (Figure 3B). Hence, we tested whether Bevacizumab may cooperate with Etoposide to eliminate the stem-like subset population of Hs294T melanoma cells. The concomitant treatment with Etoposide and Bevacizumab significantly reduced the ability of P0 melanospheres to further generate P1 spheres, although Bevacizumab alone was found partially active in P1 sphere reduction (Figure 3C). Etoposide plus Bevacizumab and also Bevacizumab added on silenced VEGF-R2 cells, are effective in inducing apoptotic death. Also, Bevacizumab administered as single agent increases apoptotic rate of melanoma cells at a level very close to that observed using a combined in Figure 3D. We also show that Bevacizumab affected VEGF signaling by reducing VEGF-R2 phosphorylation. Various studies have shown that many anti-cancer drugs kill susceptible cells by inducing apoptosis, although is well known that melanoma cells are highly resistant to anti-apoptotic drugs [35–37].

### CD133+/VEGF-R2+ Hs294T cell subset identified by cytofluorymetric analysis

**Figure 4:**
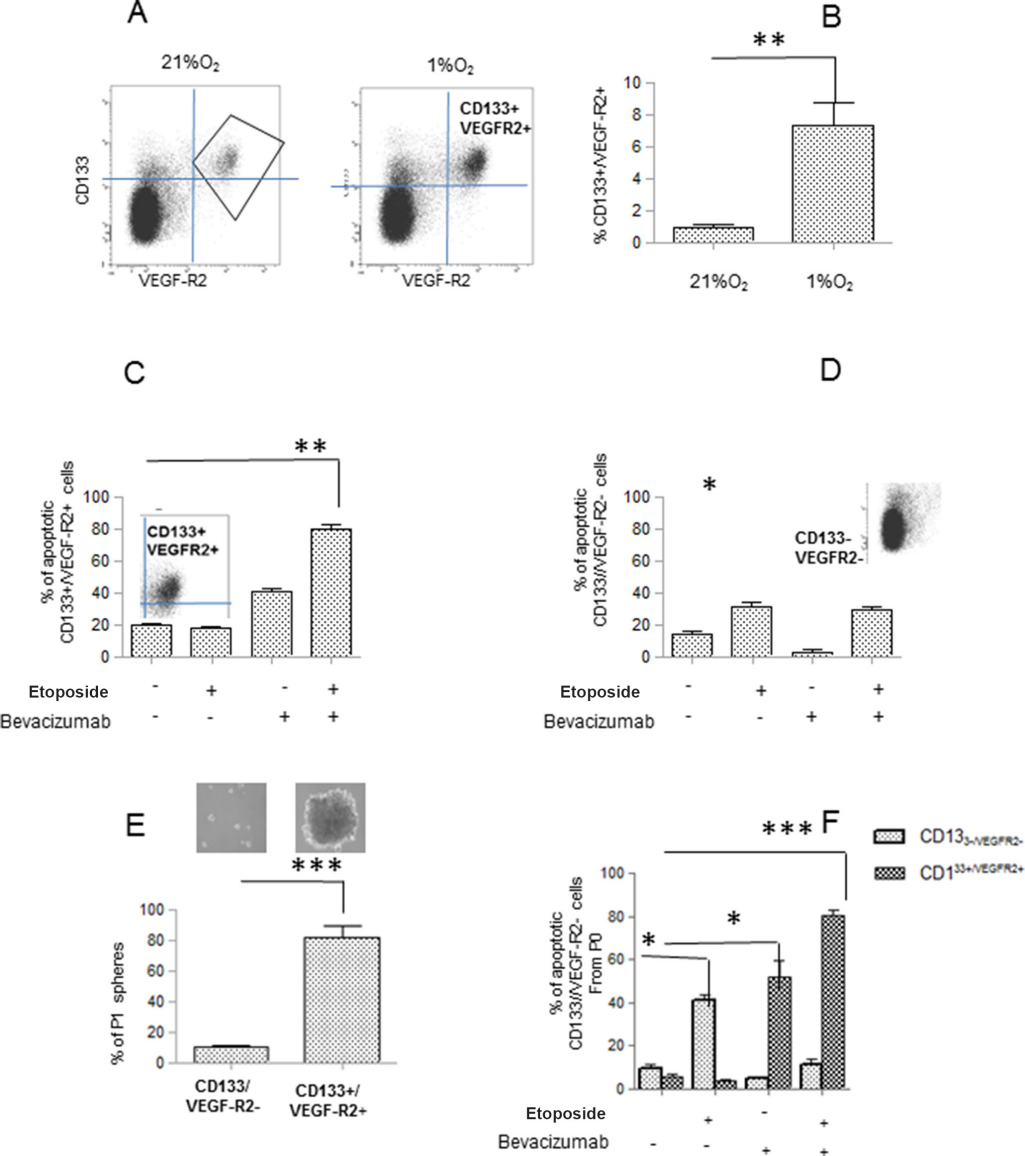
Etoposide and/or Bevacizumab treatment eradicated VEGFR-2+/CD133+ subpopulations (**A**, **B**) Cell sub populations derived from Hs294T cells cultured under normoxic or hypoxic conditions for 24 hours, were analyzed by FACS for double VEGFR-2+ and CD133+ co-expression. (**C, D**) VEGFR-2+/CD133+*(left)* and CD133-/VEGF*-(right)* gated cells were analyzed for apoptosis rate evaluated by annexin V /Propidium iodide staining. (**E**) VEGFR-2+/CD133+ were isolated by cell sorting and used to perform a melanosphere (P0 and P1) formation assay. Photographs of P0 spheres were taken and shown (above the corresponding treatment), while P1 spheres were counted and plotted. (**F**) P0 spheres derived from sorted VEGFR-2+/CD133+ and CD133-/VEGF- were treated with Etoposide (50 μM) and/or Bevacizumab (250 ng/ml) and apoptosis rate was evaluated by Annexin V/propidium iodide staining.

We performed a cytofluorymetric analysis which allowed us to identify a sub-population of cells positive for both VEGF-R2 and CD133 (CD133+ /VEGF-R2+ cells), and these cells arise with a frequency ranging from 0.6% in normoxia to 5–7% under hypoxia. As indicated by the plot reported in Figure 4A, the subpopulation VEGFR2+ is all included in the CD133+. Data revealed that CD133+ subpopulation increases under hypoxia and it acquires VEGFR2 expression (Figure 4A, 4B). When we analyzed double VEGF-R2+/CD133+ cells for Annexin V expression, we found that Etoposide/Bevacizumab treatment specifically targets these cells, as indicated by the high percentage of AnnexinV+ cells of VEGF-R2+/CD133+ subpopulation. On the other hand, Etoposide results ineffective on this subpopulation of cells (Figure 4C). Data revealed that Etoposide but not Bevacizumab is effective in inducing apoptosis of VEGF-R2 −/CD133 - subpopulation (Figure 4D). These data indicated that VEGF-R2 conferred high intrinsic resistance to Etoposide under hypoxic condition and that this resistance could be reverted by action of Bevacizumab or by neutralization VEGF-R2.

Thereafter, we sorted VEGF-R2+/CD133+ cells from hypoxic Etoposide-treated melanoma cells, a condition able to reveal higher expression of VEGF-R2 and CD133 in melanoma cells, and, upon evaluation melanosphere forming ability, we define that these cells formed more P1 spheroids (Figure 4E). In CD133+/VEGF-R2+ cells, sorted by hypoxic-Etoposide cells and exposed to Etoposide and/or Bevacizumab, high apoptotic rate correlates with Bevacizumab and more importantly with the combined treatment (Figure 4E), suggesting that Bevacizumab represents a crucial requirement for apoptosis of VEGF-R2+/CD133+ cells.

A combined treatment with Etoposide and Bevacizumab inhibits invasiveness and lung colonization of hypoxic Hs294T stem-like melanoma subset.

**Figure 5:**
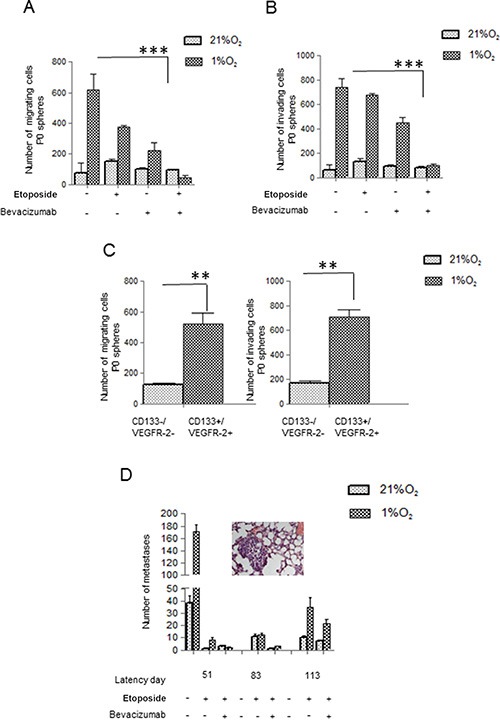
Bevacizumab reduces Hs294T cell motility and *in vivo* metastases formation (**A, B**) P0 spheres from Hs294T, treated with or without Etoposide/Bevacizumab (50 μM) and/or Bevacizumab (250 ng/ml), were disaggregated and cells were analyzed for migration *(left)* and invasion *(right*) assay by Boyden Chamber. (**C**) CD133+/VEGF-R2+ and CD133−/VEGF-R2- sub population were analyzed for cell migration and invasion. (**D**) Hs294T cells were treated in hypoxic condition with Etoposide (50 μM) alone or with Etoposide (50 μM) plus Bevacizumab (250 ng/ml) for 24 h. 1 × 10^6^ melanoma cells treated as above were injected into the lateral tail vein of SCID bg/bg mice (*n* = 5 per group) and number of lung macro and micrometastasis was evaluated at different times from injection (e.g. latency). Lungs were inspected for macrometastases using dissecting microscope, and then fixed overnight at 4C in formalin (5% in PBS) for histological analyses of micrometastases photo of micrometastasis of mice injected with hypoxic Hs294T cells treated with Etoposide. Untreated mice were used as control and they all died at the 51st day of observation **p < 0.001. **p < 0.001, ***p < 0.0001, *n* = 3.

To confirm the aggressive behavior of cells derived from P0 spheres, we tested Hs294T and A375 cells using both two (2D)- and three (3D)-dimensional migration assay. Cells derived from disaggregated hypoxic P0 spheres showed an enhanced migratory ability, and Etoposide/Bevacizumab treatment abolishes this capacity (Figure 5A–5B). Our data also demonstrated that VEGF-R2+/CD133+ subpopulation, sorted from hypoxic Etoposide treated cells, expresses a higher migration and invasiveness measured in Boyden chambers (Figure 5C). Thus, VEGF/VEGF-R2 axis associates with the migratory phenotype expressed by melanoma stem-like subset.

Overall, *in vitro* analyses indicate that Bevacizumab cooperates with Etoposide in eradicating VEGF-R2+/CD133+ cells. Thus, we injected normoxic and hypoxic Hs294T melanoma cells treated with Etoposide or Etoposide/Bevacizumab into the tail vein of SCID bg/bg mice to verify cloning efficiency. Animals were sacrificed at different time points and lungs were inspected for macro and micro-metastases (see an E&E-stained lung section showing a micrometastasis). Mice injected with Etoposide-treated hypoxic cells reveal an increased number of metastases, starting from day 51, whereas addition of Bevacizumab to Etoposide retards appearance of lung lesions until day 113, indicating a 2-fold latency period for lung colonies development following combined treatment, and reduces their number (Figure 5D). A latency period of two months in mice corresponds approximately to 5.76 years in humans [38].

## DISCUSSION

Melanoma contain a subpopulation with stem cell properties defined CSCs and a bulk of more differentiated tumor cells. CSC reflect the stage of tumor progression and express several abilities, including sphere formation. Sphere forming ability was initially observed in cultured neural stem cells [39], after that was found characterize stem cells of a variety of human cancers, suggesting that sphere forming capability represents a common growth characteristic of tumor stem cells [40]. In 2009, Singh et al. demonstrated that neurosphere-derived tumor cells expressing the neuronal stem cell surface marker Prominin-1/AC133/CD133 had an increased capacity for self-renewal and proliferation. Only the CD133+ subfraction of brain tumor cultures demonstrated the capacity to proliferate and generates neurospheres *in vitro* [41]. Among the several microenvironmental aspect of tumors, it is recognized that hypoxia drives aggressiveness and stemness in tumor cells [42–44]. Indeed, local oxygen concentration can directly influence stem cell renewal and differentiation. Stem cells might benefit from residing in hypoxic niches where oxidative DNA damage is reduced. Direct measurement of oxygen levels has revealed that bone marrow is in general quite hypoxic (1–2% O_2_) and HIF-1α transcription factor plays a major role [45, 46]. Among HIF-1α targets there is the ABC glycoprotein transporter MDR1, which confers multidrug resistance on a variety of cancer cells [46]. Indeed, the ABC transporter, Bcrp/ABCG2, is implicated in chemotherapeutic drug resistance in breast cancers [47].

Our study shows that hypoxic melanoma cells, either Hs294T or A375, isolated from P0 spheres express an high capacity to induce P1 spheres, level of CD133 and higher ability of 3D invasiveness and 2D motility also in the presence of Etoposide, a pro-apoptotic agent active on topoisomerase II [48]. High grade of motility was found by Moriyama et al. in CD133+/CXCR4+ tumor cells expressing a high metastatic potential [49]. We also demonstrated that Etoposide-exposed cells isolated from P0 spheres express high level of VEGF-R2 and treatment with siRNA for VEGF-R2 or Bevacizumab abolishes P1 sphere formation. Furthermore, Etoposide Bevacizumab cooperation promotes apoptosis in cells derived from P0 spheres. It is of importance to note, that also Bevacizumab, as a single agent is partially active in the reduction of P1 sphere formation and apoptosis induction. Hypoxia and VEGF-R2 expression have been also correlated with uveal melanoma aggressiveness and stem-like behavior [50–51].

In addition to melanoma, the VEGF/VEGF-R2 axis is involved in tumor progression of acute myeloid leukemia, glioma, breast and ovarian cancer [52–54]. Bevacizumab has already been approved in combination with chemotherapeutic agents for the treatment of metastatic colorectal cancers (21, 22). Metronomic chemotherapy with daily oral administration of Etoposide plus Bevacizumab was used for treatment of malignant glioma [55].

Furthermore, when, by flow cytometry analysis, we analyzed VEGF-R2+/CD133+cells for Annexin V expression, we found that the combined Etoposide and Bevacizumab treatment specifically targets these cells. Then, CD133+/VEGF-R2+ cells treated with Etoposide and Bevacizumab were sorted and found Annexin V positive. Thus, Bevacizumab cooperates with Etoposide in eradicating VEGF-R2+/CD133+ cells. Melanoma cells with a stem-like phenotype are likely to achieve a drug-resistant phenotype, including to apoptotic agents, such Etoposide. However, when the VEGF/VEGFR2 axis will be inhibited in these cells, it is possible that cells regain apoptotic sensitivity to Etoposide.

To demonstrate the effectiveness of this cooperation, we verified cloning efficiency of hypoxic Hs294T melanoma cells treated with Etoposide and/or Bevacizumab injected into tail vein of immunodeficient mice. We observed an increase latency and a reduced lung colonization of Etoposide/Bevacizumab treated sub-population. The occurrence of lung metastases even after a long period in animals injected with melanoma cells treated *in vitro* with Etoposide and Bevacizumab might be ascribed to an acquired escaping ability to apoptosis of treated CSCs. However, considering that melanoma cells were exposed to a single treatment before *in vivo* injection, we may consider the possibility that a multiple and adequate schedule of exposure might be instrumental in reducing relapse.

In conclusion, we identified a stem-like cell subpopulation from hypoxic Hs294T and A375 human melanoma cells, which is specifically targeted *in vitro* by Etoposide and Bevacizumab co-treatment. We therefore speculate that Etoposide/Bevacizumab treatment could have a role in the control of stem-like subset of melanoma.

## MATERIALS AND METHODS

Unless specified all reagents were obtained from Sigma. Hs294T and A375 cells were from ATCC, VEGFR2 and pVEGFR2 antibodies for Western Blotting were from Santa Cruz Biotechnology. Transwell system were from Costar. Low attachment 100 mm plate were from Corning.VEGFR-2/KDR-APC (clone:ES8-20E6) and CD133/2 (293C3-PE), clone: 293C3 were from Miltenyi. PVDF for Western Blot analysis was from Millipore. siRNA oligonucleotides targeting VEGF-R2 protein were obtained from Ambion (ID 221 and ID 222). Annexin-V fluorescein isothiocyanate apoptosis kit was from Roche Diagnostic.

### Cell cultures and transfections

Hs294T and A375 melanoma cells were cultured in Dulbecco Modified Eaglel Medium (DMEM) supplemented with 10% fetal bovine calf serum (FCS), in 5% CO_2_ humidified atmosphere. Experiments under hypoxic condition (1% O_2_) were performed in the hypoxic incubator. For transient transfections, Hs294T cells were plated in six-well plate cell culture dishes and grown to 70% confluence. The siRNA was diluted to a final concentration of 20 nM. Transfections were performed 48 h before the indicated treatment using Lipofectamine (Invitrogen), following manufacturer's recommendations. After 48 hours from trasfection cell were treated as indicated conditions reported in figure legends.

### Flow cytometric analysis and cell sorting

#### Apoptotic assay

Annexin-V staining was performed using the Annexin-V fluorescein isothiocyanate apoptosis kit according to the manufacturer's instructions. Gated cells were plotted on a dot-plot showing Annexin-V staining and propidium iodide (PI) staining. Index of apoptotic cells was determinate adding Annexin V positive (early apoptotic) cells to Annexin/PI positive ones (late apoptotic).

#### Analysis of CD133 and VEGFR2 marker

To analyze the expression of VEGFR2 and CD133 in A375 and Hs294T cell lines, cells were routinely cultured, harvested, trypsin-digested and re-suspended in stain buffer (1 × 10^6^ cells in 80 μl). Cells were then treated with 20 μl FcR Blocking Reagent for 15 min, and steined with antibodies anti-CD133-(PE), and VEGFR2 (APC) both diluited (1:11) for 30 min. After staining, cells were subjected to flow cytometry for analysis using BD FACS CANTO II.

#### Cell sorting

To isolate CD133^+^/VEGFR2+ populations in the Hs294T line, cells were digested with Accutase and blocked with FcR Blocking Reagent. Propidium iodide staining was applied to exclude the dead cells. Live cells were incubated with antibodies. Anti-CD133 and antiVEGF-R2 were incubated for 30 min and cells were sorted by a cell sorter BD FACSARIA III, CA.

#### Western blot analysis

1 × 10^6^ Hs294T cells derived from different experimental conditions were lysed for 20 min on ice in 500 μl of complete RIPA lysis buffer (50 mM Tris-HCl, pH 7.5, 150 mM NaCl, 1% Nonidet P-40, 2 mM EGTA, 1 mM sodium orthovanadate, 1 mM phenyl-methanesulphonyl-fluoride, 10 μg/ml aprotinin, 10 μg/ml leupeptin). Lysates were separated by SDS/PAGE, and transferred into PVDF. Immunoblots were incubated in 3% BSA, 10 mM Tris/HCl (pH 7.5), 1 mM EDTA, and 0.1% Tween 20, for 1 h at room temperature, probed first with specific antibodies p VEGF-R2 and VEGF-R2 (1:1000) and then with secondary antibodies (1:500).

#### RT-PCR analysis

Total RNA from monolayers or melanospheres of A375 and Hs294T melanoma cells was extracted using RNeasy (Qiagen) according to the manufacturer instructions. Strands of cDNA were synthesized using a high capacity cDNA reverse transcription kit (Applied Biosystem) using 1 μg of total RNA. For quantification of mRNA expression, Real-Time PCR, using Power SYBR green dye (Applied Biosystem) was done on a 7500 Fast Real Time PCR system (Applied Biosystem). The primers were NANOG: 5′-ACCTTGGCTGCCGTCTCTGG-3′ (forward), 5′-AGC AAAGCCTCCCAATCCCAAACA-3′ (reverse); KLF4: 5′-GCAGCCACCTGGCGAGTCTG-3′ (forward), 5′-CCG CCAGCGGTTATTCGGGG-3′ (reverse); SOX2 5′-GAG CTTTGCAGGAAGTTTGC-3′ (forward), 5′-GCAAGAA GCCTCTCCTTGAA-3′ (reverse); OCT4: 5′-TTTTGGTA CCCCAGGCTATG-3′ (forward), 5′-GCAGGCACCTCAG TTTGAAT-3′ (reverse), VEGFR2 GCTTTGGCCCAA TAATCAGA (forward), ACACGACTCCATGTTGGTCA (reverse). Data were normalized to those obtained with Glyceraldehyde-3-phosphate deydrogenase primers. Results (mean ± SD) are the mean of three different experiments.

#### Melanospheres formation and treatment

To examine the effects of different agents on melanosphere formation, cells were detached using Accutase (Sigma) and plated at 1000 cells/cm^2^ on low attachment 100 mm plate in serum-free DMEM/F12 1:1 (Invitrogen, Carlsbad, CA, USA) supplemented with N2 (Invitrogen), 0.6% glucose, 20 μg/ml insulin, 10 ng/ml b-FGF and 10 ng/ml EGF. Cells were grown under these conditions for 15 days to obtain P0 melanospheres. For the evaluation of self-renewal, a single melanosphere was dissociated in single cells with Accutase, and a diluition of 1 cell/well into 96-well low attachment plates was performed in order to isolate individual P1 melanospheres. After about 10 days single-cell cloning was confirmed by microscopic analysis, and single clones were counted. P1 spheres were then incubated for 5 days in the absence (controls) or presence of testing agent(s).

#### Migration and invasiveness assays

Migration and invasiveness of Hs294T and A375 cell were determined using the Transwell system, equipped with polyvinylpyrrolidone-free polycarbonate 8-μm pore-size filters (diameter, 13 mm). Migration or invasiveness assays are distinguished by absence (migration assay) or presence (invasiveness assay) of a 3D barrier of Matrigel (BD Biosciences). Matrigel was diluted (30 μg in 100 μl of H_2_O), added to top chamber, allowed to solidify for 1 h at 37°C, and air dried for 16 h. Matrigel barrier was reconstituted with 100 μl of Dulbecco's modified Eagle's medium for 2 h at 37°C before use. Cells were loaded into the upper compartment (5 × 10^4^ cells in 200 μl) in serum-deprived growth medium. Transwell (coated or not with Matrigel) were placed into 24-well culture dishes containing 500 μl of Dulbecco's modified Eagle's medium enriched with 10% serum as a chemoattractant and incubated at 37°C for 24 h, under hypoxic or normoxic condition. Non-invading cells were mechanically removed using cotton swabs, and the microporous membrane containing the invading cells was fixed in 96% methanol and stained with Diff-Quick staining solution. Invasiveness were evaluated counting migrated cells to the lower surfaces of polycarbonate filters. Numbers of cells in six randomly chosen fields were determined for each filter, and means ± SD was plotted.

#### Lung colonization assay

Experiments were conducted in accordance with national guidelines and were approved by the ethics committee of the Animal Welfare Office of the Italian Work Ministry and conformed to the legal mandates and Italian guidelines for the care and maintenance of laboratory animals. 2 × 10^6^ Hs294T melanoma cells either treated with Etoposide or Etoposide plus Bevacizumab and suspended in 0.2 ml of PBS were injected into the lateral tail vein of six- to eight-week old female SCID-bg/bg mice (Charles River Laboratories International, Wilmington, MA, USA) (five animals per group). Animals were monitored every 3 days and sacrificed at different time points. Lungs were inspected for metastatic nodules with the help of a dissecting microscope, and then fixed overnight in 5% formalin in PBS for histological analysis.

#### Statistical analysis

Data are presented as means ± SD from at least three independent experiments. Statistical analysis of the data was performed using two way Anova Bonferroni Test, and *p* value of ≤ 0.05 is considered statistically significant.
